# Draft Genome Sequence of *Enterobacter* sp. Strain AD2-3, Isolated from a Postmining Site in Benguet, Philippines

**DOI:** 10.1128/MRA.00563-19

**Published:** 2019-07-25

**Authors:** Jocelyn T. Zarate, Genevieve Mae B. Aquino, Joana Marie C. Cruz, Neilyn O. Villa, Albert Remus R. Rosana

**Affiliations:** a National Institute of Molecular Biology and Biotechnology, University of the Philippines Los Baños, Laguna, Philippines; b Office of the Vice-Chancellor for Research and Extension, University of the Philippines Los Baños, Laguna, Philippines; c Genetics and Molecular Biology Division, Institute of Biological Sciences, University of the Philippines Los Baños, Laguna, Philippines; d Department of Chemistry, University of Alberta, Edmonton, Alberta, Canada; University of Southern California

## Abstract

The novel strain Enterobacter sp. strain AD2-3 was isolated from postmining soil samples collected from Antamok mine in Benguet, Philippines. Here, we report a draft of its whole-genome sequence, with predicted gene inventories supporting metal tolerance, nitrogen fixation, phosphate solubilization, and indole acetic acid production.

## ANNOUNCEMENT

Various Enterobacter species with plant probiotic properties have been isolated (1), including those from the rhizosphere of economically important crops (2). Here, we report the draft genome sequence of *Enterobacter* sp. strain AD2-3, isolated from soil samples collected at a post-gold mine site (Antamok) in Benguet, Philippines. AD2-3 was sequenced because it is one of the components of a microbial inoculum under the trademark Anfer (Antamok biofertilizer) being developed as part of the MykoPlus biofertilizer technology (http://www.pcaarrd.dost.gov.ph/home/portal/index.php/quick-information-dispatch/2435-beneficial-microorganisms-makes-soil-healthier-and-increases-yield) for the revegetation of post-gold mine sites in the Philippines (3, 4). The soil sample was collected at 16°23′58″N, 120°39′45″E at a depth of 5 to 10 cm along the road and not attached to any plant or root system.

AD2-3 was isolated by spreading 0.1 ml of diluted soil solution on Ashby-sucrose agar (5), mannitol agar (6), and Burk’s medium (7), and each was incubated at 28 to 30°C for 2 to 3 days. The colonies of bacteria that grew on all media were purified on Dobereiner’s medium (8, 9), followed by National Botanical Research Institute Phosphate growth medium (10) for 2 to 3 days of incubation each at 28°C. Single-colony isolates were streaked for at least 5 rounds of purification, generating isolate AD2-3. AD2-3 was grown in 3 ml nutrient broth at 28°C with shaking at 120 rpm for 16 h, and genomic DNA (gDNA) was purified using the KingFisher cell and tissue DNA kit (Thermo Fisher Scientific, Waltham, MA), according to the manufacturer’s protocol. The DNA library was prepared using a Nextera XT library kit (Illumina, San Diego, CA) and sequenced on the MiSeq platform using the 600-cycle V3 reagent kit at the Philippine Genome Center. Quality checking was performed with FastQC version 0.11.7 and adapter trimming with Trimmomatic version 0.36.5 (11). The trimmed reads were *de novo* assembled using SPAdes version 3.13.0 (12). Gene prediction was performed using Prokka version 1.13 (13) and the NCBI PGAP version 4.8 (14). Genus identity was determined by BLAST (15) alignment of the predicted 16S rRNA gene sequence against the SILVA database (16). The genome-wide average nucleotide identity (gANI) and alignment fraction (AF) using the Microbial Species Identifier (MiSI) calculator employed in IMG/M-ER (17) were used to determine the relatedness of AD2-3 to the endophytic *Enterobacter* sp. strain DC3 (18), the most similar genome identified by LASTZ version 1.3.2 (19). Strain novelty was verified by the digital DNA-DNA hybridization (dDDH) score using the Genome-to-Genome Distance Calculator version 2.1 (20). All programs were run with default parameters, unless otherwise noted.

The AD2-3 genome has an ANI of 94.27% (AF, 0.89) and dDDH of <70% with the DC3 genome, whereas the most closely related type strain, *E. asburiae* ATCC 35953, has an ANI of 94% (AF, 0.58). Paired-end sequencing yielded 627,045 reads (22× coverage). The draft genome is 4,639,072 bp in 16 contigs (*N*_50_, 574,736 bp) and has a G+C content of 55%. Genome annotation detected 4,293 coding sequences, 13 rRNA genes, 65 pseudogenes, and 74 tRNAs. The genome contains gene inventories supporting rhizosphere processes and having plant growth-promoting properties (21–23). An aryl polyene cluster for protection against reactive oxygen species (24), a homoserine lactone cluster for quorum sensing (25), and genes for Fe(II/III) (*efe*) acquisition (26) were identified. Genes were also detected for indole acetic acid production (*iaa* and *ipdC*), phosphate solubilization (*bgl* and *ybg*), and nodulation and atmospheric nitrogen fixation (*nif* and *nod*) (1).

### Data availability.

The raw reads were deposited at the NCBI Sequence Read Archive with accession number SRR8723068. The assembled draft genome sequence was deposited in DDBJ/ENA/GenBank under accession number SOPQ00000000. The version described in this paper is the first version.

## References

[1] Carro L, Nouioui I 2017 Taxonomy and systematics of plant probiotic bacteria in the genomic era. AIMS Microbiol 3:383–412. doi:10.3934/microbiol.2017.3.383. 31294168 PMC6604993

[2] Gupta A, Gopal M, Thomas GV, Manikandan V, Gajewski J, Thomas G, Seshagiri S, Schuster SC, Rajesh P, Gupta R 2014 Whole genome sequencing and analysis of plant growth promoting bacteria isolated from the rhizosphere of plantation crops coconut, cocoa and arecanut. PLoS One 9:e104259. doi:10.1371/journal.pone.0104259. 25162593 PMC4146471

[3] Zarate JT 2017 Characterization of soil bio-physico-chemical properties and collection of beneficial soil microorganisms potential to restore post-gold-mining areas in Indonesia and the Philippines. Terminal report SEAMEO BIOTROP, Bogor, Indonesia.

[4] Zarate JT 2018 Increasing the adaptation of MykoPlus technology through farmer managed practices. Annual reports BIOTECH, Los Baños, Philippines.

[5] Jiménez DJ, Montaña JS, Martinez MM 2011 Characterization of free nitrogen fixing bacteria of the genus Azotobacter in organic vegetable-grown Colombian soils. Braz J Microbiol 42:846–858. doi:10.1590/S1517-83822011000300003. 24031700 PMC3768769

[6] Bhaduri J, Kundu P, Kabir SE, Roy SK 2016 Isolation and characterisation of non-symbiotic nitrogen fixing bacteria (*Azotobacter* sp.) from tea field soil of Terai region of North Bengal, India. Int J Adv Res Eng Appl Sci 5:19–33.

[7] Nagalla AD, Herath JV, Suriyagoda LDB 2017 Evaluating the growth performance of two *Azotobactor* spp. in liquid glucose broth and tryptic peptone broth as inoculum for the production of bio-fertilizers. Trop Agric Res 28:312–318. doi:10.4038/tar.v28i3.8234.

[8] Zarate JT, Trinidad LC, Alfafara C, Lat E 2014 Optimization and scale up production of MykoPlus for the biofertilizer and bio-organic fertilizer industries. Terminal report BIOTECH, Los Baños, Philippines.

[9] Zarate JT, Trinidad LC, Chico MLM, Luar LR, Gabo PJ, Santiago MJ 2015 MykoPlus technology promotion and demonstration trials for corn (*Zea mays*) and eggplant (*Solanum melongena*). Terminal report BIOTECH, Los Baños, Philippines.

[10] Kapri A, Tewari L 2010 Phosphate solubilization potential and phosphatase activity of rhizosphere *Trichoderma* spp. Braz J Microbiol 41:787–795. doi:10.1590/s1517-83822010005000001. 24031556 PMC3768635

[11] Bolger AM, Lohse M, Usadel B 2014 Trimmomatic: a flexible trimmer for Illumina sequence data. Bioinformatics 30:2114–2120. doi:10.1093/bioinformatics/btu170. 24695404 PMC4103590

[12] Bankevich A, Nurk S, Antipov D, Gurevich AA, Dvorkin M, Kulikov AS, Lesin VM, Nikolenko SI, Pham S, Prjibelski AD, Pyshkin AV, Sirotkin AV, Vyahhi N, Tesler G, Alekseyev MA, Pevzner PA 2012 SPAdes: a new genome assembly algorithm and its applications to single-cell sequencing. J Comput Biol 19:455–477. doi:10.1089/cmb.2012.0021. 22506599 PMC3342519

[13] Seemann T 2014 Prokka: rapid prokaryotic genome annotation. Bioinformatics 30:2068–2069. doi:10.1093/bioinformatics/btu153. 24642063

[14] Tatusova T, DiCuccio M, Badretdin A, Chetvernin V, Nawrocki EP, Zaslavsky L, Lomsadze A, Pruitt KD, Borodovsky M, Ostell J 2016 NCBI Prokaryotic Genome Annotation Pipeline. Nucleic Acids Res 44:6614–6624. doi:10.1093/nar/gkw569. 27342282 PMC5001611

[15] Boratyn GM, Camacho C, Cooper PS, Coulouris G, Fong A, Ma N, Madden TL, Matten WT, McGinnis SD, Merezhuk Y, Raytselis Y, Sayers EW, Tao T, Ye J, Zaretskaya I 2013 BLAST: a more efficient report with usability improvements. Nucleic Acids Res 41:W29–W33. doi:10.1093/nar/gkt282. 23609542 PMC3692093

[16] Quast C, Pruesse E, Yilmaz P, Gerken J, Schweer T, Yarza P, Peplies J, Glöckner FO 2012 The SILVA ribosomal RNA gene database project: improved data processing and Web-based tools. Nucleic Acids Res 41:D590–D596. doi:10.1093/nar/gks1219. 23193283 PMC3531112

[17] Varghese NJ, Mukherjee S, Ivanova N, Konstantinidis KT, Mavrommatis K, Kyrpides NC, Pati A 2015 Microbial species delineation using whole genome sequences. Nucleic Acids Res 43:6761–6771. doi:10.1093/nar/gkv657. 26150420 PMC4538840

[18] Gan HM, Triassi AJ, Wheatley MS, Savka MA, Hudson AO 2014 High-quality draft whole-genome sequences of three strains of *Enterobacter* isolated from Jamaican *Dioscorea cayenensis* (yellow yam). Genome Announc 2:e00170-14. doi:10.1128/genomeA.00170-14. 24625871 PMC3953192

[19] Harris RS 2007 Improved pairwise alignment of genomic DNA. PhD thesis. Pennsylvania State University, State College, PA.

[20] Meier-Kolthoff JP, Auch AF, Klenk HP, Göker M 2013 Genome sequence-based species delimitation with confidence intervals and improved distance functions. BMC Bioinformatics 14:60. doi:10.1186/1471-2105-14-60. 23432962 PMC3665452

[21] Singh M, Chaudhary S, Sareen D 2017 Non-ribosomal peptide synthetases: identifying the cryptic gene clusters and decoding the natural product. J Biosci 42:175–187. doi:10.1007/s12038-017-9663-z. 28229977

[22] Schwalen CJ, Hudson GA, Kille B, Mitchell DA 2018 Bioinformatic expansion and discovery of thiopeptide antibiotics. J Am Chem Soc 140:9494–9501. doi:10.1021/jacs.8b03896. 29983054 PMC6070396

[23] Saha M, Sarkar S, Sarkar B, Sharma BK, Bhattacharjee S, Tribedi P 2016 Microbial siderophores and their potential applications: a review. Environ Sci Pollut Res 23:3984–3999. doi:10.1007/s11356-015-4294-0. 25758420

[24] Schöner TA, Gassel S, Osawa A, Tobias NJ, Okuno Y, Sakakibara Y, Shindo K, Sandmann G, Bode HB 2016 Aryl polyenes, a highly abundant class of bacterial natural products, are functionally related to antioxidative carotenoids. Chembiochem 17:247–253. doi:10.1002/cbic.201500474. 26629877

[25] Ma H, Ma S, Hu H, Ding L, Ren H 2018 The biological role of N-acyl-homoserine lactone-based quorum sensing (QS) in EPS production and microbial community assembly during anaerobic granulation process. Sci Rep 8:15793. doi:10.1038/s41598-018-34183-3. 30361560 PMC6202354

[26] Roy EM, Griffith KL 2017 Characterization of a novel iron acquisition activity that coordinates the iron response with population density under iron-replete conditions in *Bacillus subtilis*. J Bacteriol 199:e00487-16. doi:10.1128/JB.00487-16. 27795321 PMC5165090

